# Level of Anxiety Caused by the Coronavirus (COVID-19) Pandemic among Dentists in Poland

**DOI:** 10.3390/medicina58030415

**Published:** 2022-03-11

**Authors:** Ewa Rusyan, Agnieszka Mielczarek, Agnieszka Bogusławska-Kapała, Kamil Adamczyk, Robert Piec, Barbara Szykuła-Piec

**Affiliations:** 1 Department of Conservative Dentistry, Warsaw Medical University, 02-097 Warsaw, Poland; erusyan@wum.edu.pl (E.R.); agnieszka.mielczarek@wum.edu.pl (A.M.); 2 Department of Comprehensive Dentistry, Warsaw Medical University, 02-097 Warsaw, Poland; aboguslawska@wum.edu.pl; 3 Central Clinical Hospital of the Ministry of Interior and Administration in Warsaw, 02-507 Warsaw, Poland; kamil.adamczyk@cskmswia.gov.pl; 4 The Main School of Fire Service, 01-629 Warsaw, Poland; rpiec@sgsp.edu.pl

**Keywords:** COVID-19, dentistry, anxiety, fear

## Abstract

*Background and Objectives*: The early information on both the speed and high morbidity rate and, above all, mortality triggered the symptoms of COVID-19-related panic and anxiety. Dentists were listed in the top five professions with the highest risk of transmission of the virus. The aim of the present study was to investigate the correlation between the fear level of COVID-19 and sociodemographic variables in Polish dentists. *Materials and Methods*: A cross-sectional study was conducted via an online survey questionnaire with seven statements in the COVID-19 Fear Scale (FCV-19S). The online questionnaire was completed by 356 dentists. The SPSS and PQStat were used to analyze, validate, and assess correlations and logistic regression. *Results*: In the studied population of dentists, the perceived level of anxiety associated with COVID-19 should be considered relatively low. When the respondents had children, lived with the elderly, or looked after them, the observed level of anxiety was higher, and physical symptoms, such as sweating palms and increased heart rate, occurred. *Conclusions*: Studies concerning the anxiety level related to COVID-19 carried out among Polish dentists ascertained that the tested level of anxiety among dentists was relatively low. The COVID-19 Fear Scale (FCV-19S) adjusted to the Polish language requirements is a reliable tool that can be used effectively for analyzing the impact of any pandemic on the Polish-speaking population.

## 1. Introduction

One of the global challenges that emerged worldwide at the end of 2019 was the coronavirus (COVID-19) pandemic. Early information on both the speed and high morbidity rate and, above all, mortality triggered the symptoms of COVID-19-related panic and anxiety [1]. Under these conditions, working in occupations exposed to infection has become particularly stressful. According to the New York Times analysis based on data of the U.S. Department of Labor [2], this group includes dentists listed in the top five professions with the highest risk of transmission of the virus. In the first phase of the spread of the disease, most clinics in Poland suspended their practice. At the beginning of the pandemic in Poland, only departments working in emergency provided services to patients with acute conditions [3]. Insufficient supply of personal protective equipment as well as the lack of clear legal interpretations in the field of civil and criminal liability caused anxiety as to the resumption of operations.

Anxiety and fear are universal basic emotions that a person can experience, especially in difficult, unpredictable situations, where common sense is replaced by fear and speculation, e.g., due to the lack of reliable and verified information about potential danger. As of 9 July 2020, nearly 12 million confirmed COVID-19 cases were reported to the WHO worldwide, including over 540,000 cases of deaths [4]. The rate of spread of the virus and the high global COVID-19 mortality rate may explain the fear of encountering potentially infected people [5]. Multiple studies confirm the strong impact that the threat of the new coronavirus disease (COVID-19) has on individuals [6,7]. Such strong emotions trigger both physiological and psychological reactions. At present, due to the prolonged state of the epidemic, it is important to pay attention to the psychological sphere [8], which is not insignificant for the transmission of the virus. Fear, defined as an “unclear, unpleasant emotional state characterized by experiencing fear, stress and annoyance, along with anxiety”, says Taylor, is the main reaction that strongly influences the perception of the environment and individual behavior [9]. 

Pandemics are large-scale epidemics that spread around the world. In recent years, virologists have been outdoing each other in reports and predictions about future pandemics with probable, catastrophic consequences for humanity. In Poland, as of 9 July 2020, we had over 35,000 confirmed cases of COVID-19 with 1542 people who died due to complications from the coronavirus infection. With these statistics, most of the restrictions were lifted while concurrently maintaining imposing the necessity of wearing a mask in public places and keeping the required social distance. This study was generally aimed at investigating the association between the fear level of COVID-19 and sociodemographic variables in Polish dentists.

## 2. Materials and Methods

### 2.1. Study Design, Setting, Participants

In the study, a tool developed by the team of Ahorsu et al. was used, The COVID-19 Fear Scale (FCV-19S) [10], translated into Polish. The FCV-19S scale was validated for use as a reliable research tool in multiple studies [11,12,13]. The English version of the scale questionnaire was translated into Polish according to generally accepted rules by two independent translators. After comparing the received texts, a version was created on the basis that, according to an expert in the field of sociology, psychology, and medical science, best reflects the original version. In order to assess the impact of the pandemic on the level of fear in the study group, a decision was made to use the FCV-19S scale as a proven research tool. After this stage, the tool was used in accordance with the research assumptions.

Data obtained as a result of the study with the use of the scale questionnaire were subjected to statistical analysis in order to assess the reliability of the developed questionnaires. All the obtained results were statistically analyzed with the PQStat 18 for Windows Software. The entire statistical analysis was performed at the confidence level of α = 0.05. The results in the case of the probability of making a type 1 error that involved rejecting the null (true) hypothesis lower than 0.05 were considered significant.

**Figure 1:**
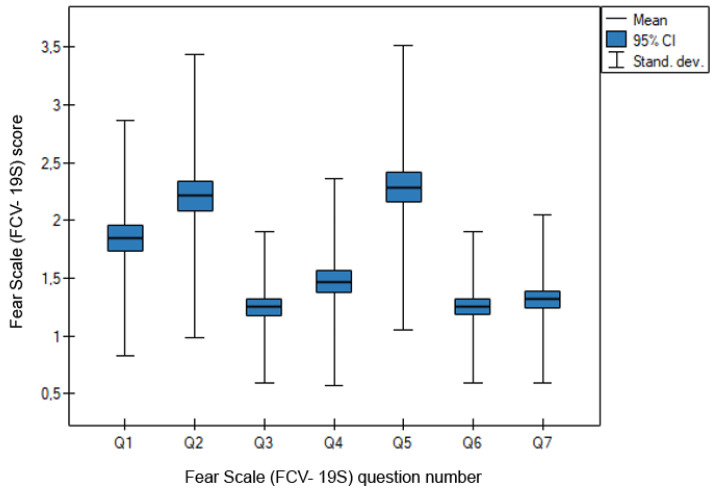
Distribution of responses in the COVID-19 Fear Scale questionnaire (FCV-19S) describing anxiety symptoms.

In this study, the internal consistency of the scales was tested using Lee Cronbach’s alpha coefficient and by determining the correlation coefficients between the answers to individual questions and the total score of the scale. Internal consistency is assumed to be good if Lee Cronbach’s alpha is at least 0.7—in the current study, the alpha is 0.9. The results are presented in Figure 1.

### 2.2. Variables

The variables were questions included in the questionnaire. The questionnaire consisted of demographic questions and were an essential part, in accordance with FCV-19S. These were 7 statements/questions meant to assess the perceived fear of COVID-19. The questions were closed ended. The level of anxiety was evaluated on a five-point scale, where the value of 1 means that a respondent did not have a given symptom, and 5 means that a respondent felt it strongly.

Patients’ fear of COVID-19 was measured by questions and statements:

1. I am afraid of coronavirus (COVID-19) more than anything else.
2. Thinking about coronavirus (COVID-19) makes me uncomfortable.
3. My palms sweat when I think about coronavirus.
4. I am worried that I will die from the coronavirus infection.
5. When I hear about coronavirus (COVID-19) in the media and on the Internet, I get nervous and worried.
6. I cannot sleep because of coronavirus.
7. My heart starts to beat faster when I think about coronavirus.

New variables were created: factor 1, emotional fear reactions (questions 1, 2, 4, and 5); factor 2, symptomatic expressions of fear (questions 3, 6, and 7); and factor 3, the general level of anxiety, consisting of all 7 symptoms of anxiety (questions 1–7). 

A cross-sectional study was conducted in the period from 26 May 2020 to 18 June 2020, with the use of an online survey questionnaire. Data were collected based on social media. In this period in Poland (from 26 May to 18 June), there were 22,000 active coronavirus cases to 30,000, with an average of 400 cases per day. The sample consisted of dentists from clinical and academic centers in Warsaw and Gdańsk, Poland. Those dentists were active professionally with available good technical support, with assured personal protective equipment and with current knowledge arising, among others, from academic lectures. Participants accessed the final questionnaire via a link in the emails and information in the Messenger application as well as via a link shared in two closed Facebook groups whose members are dentists only. Data were collected using a specially prepared Google Forms questionnaire. Consent was implied upon completion and submission of the questionnaire. Submitted surveys were collated in a directory and de-identified prior to analysis. The research has been approved by the Bioethics Committee at Medical University of Warsaw—approval number AKBE/143/2020. Written informed consent was obtained from all the participants.

### 2.3. Study Size

According to the Supreme Medical Chamber, which is a professional self-government of dentists, 37,773 people practiced this profession in Poland in 2020, including 28,458 (75%) women and 9315 (25%) men [14]. Three hundred and fifty-six people completed the online questionnaire, and three hundred and forty-seven questionnaires were classified for further analysis after the correctness of the entered data was assessed, including 71% women and 29% men. In the studied group, the proportions of men and women were similar to the general distribution characterizing the gender distribution among dentists in Poland. Only the questionnaires in which the respondent answered all questions and the marked internship was in the range from 1 to 60 were allowed for further analysis.

For the entire population of dentists, assuming a significance level 0.05 and a margin of error of 0.05, the required number of people taking the tests is 380. The collected number of questionnaires was slightly smaller, and consequently, a margin of error was calculated. For the following data:

- Confidence level (α) 95%
- Sample size (*n*) 1000
- Proportion percentage (*p*) 50
- Population size (*N*) 37,773

the obtained margin of error was: ±5.237%. 

**Figure 2:**
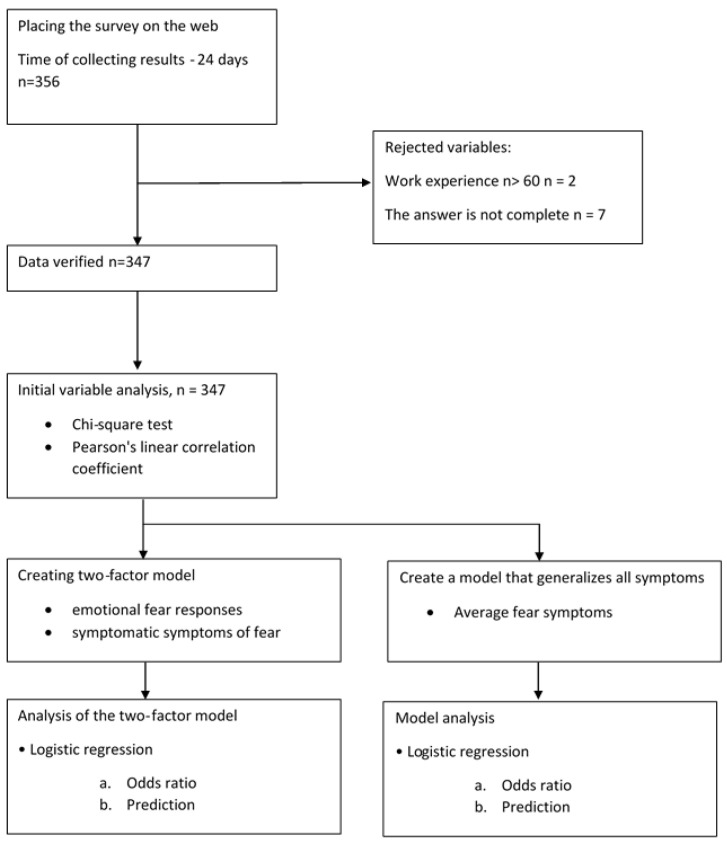
Flow diagram—the study population (respondents) and the statistical methods using.

The study population and the statistical methods are shown in Figure 2.

### 2.4. Statistical Methods

SPSS Statistics version 21.0 and PQStat were used to carry out statistical analyses, and to correlate and assess the relation of responses, among others, the chi-square test and Pearson’s correlation coefficient were used. Logistic regression analysis was performed for the newly created variables.

A two-factor model of the COVID-19 measure was built. New variables were created: factor 1, emotional fear reactions (questions 1, 2, 4, and 5), and factor 2, symptomatic expressions of fear (questions 3, 6, and 7). It was established that if the average is less than 2.5, the respondent is not afraid, and the variable takes the value 0; if the average is greater than 2.51, the respondent is afraid, and the variable takes the value 1. Additionally, a model was created to generalize all symptoms—the general level of anxiety, consisting of all 7 symptoms of anxiety (questions 1–7); analogous values were adopted if the average is less than 2.5, indicating the respondent is not afraid, and the variable takes the value 0; if the average is greater than 2.51, the respondent is afraid, and the variable takes the value 1. After constructing new variables, a logistic regression analysis was performed.

### 2.5. Quantitative Variables

The answers were coded from 1 for “definitely not” to 5 for “definitely yes”, with the possibility of marking “3” for imprecise definition of one’s opinion. The applied Likert scale allows for averaging responses in two groups of statements: 1 and 2—general low level of anxiety, 4 and 5—general high level of anxiety. In the new variables constructed based on the obtained responses, it was established that if the average is less than 2.5, the respondent is not afraid, and then, the variable acquires the value 0; if the average is greater than 2.51, the respondent is afraid, and the variable acquires the value 1.

### 2.6. Bias

The survey was voluntary and anonymous but distributed via the Internet and social media. Currently, most educated people use the Internet and social media, and yet, it cannot be ruled out that there are dentists who use the Internet very rarely. Large, renowned clinical and academic centers in Warsaw (the largest city in Poland) and Gdańsk (6th city in terms of the number of inhabitants in Poland) were used to collect the data, where even in the first wave of the COVID-19 pandemic, there was relatively high availability of personal protective equipment in comparison to small centers.

## 3. Results

### Participants

Three hundred and fifty-six people completed the online questionnaire, and three hundred and forty-seven questionnaires were classified for further analysis after the correctness of the entered data was assessed, including 71% women and 29% men. The questionnaire was completed by dentists with very little 1-year work experience and very experienced dentists with 49 years of work experience (mean 12.48 years). Detailed data are presented in Table 1.

In the studied population of dentists, the perceived level of anxiety associated with COVID-19 should be considered relatively low. When the respondents had children, lived with the elderly, or looked after them, the observed level of anxiety was higher, and physical symptoms, such as sweating palms and increased heart rate, occurred. Detailed data are presented in Table 2.

In the next step, which combined all the aforementioned symptoms, both physiological and psychological, the risk of increased anxiety was found in 8.2% of the research sample (indications 4 and 5) (Table 3).

The results presented in Table 4 indicate that gender did not have a statistically significant influence on the level of anxiety in the respondents. Nor can it be said that seniority affects the level of anxiety. However, the level of anxiety seems to be significantly influenced by having children.

Analyses of the distribution of indications and test results allow the statistically significant assumption that people with children are afraid of coronavirus more than anything else. The statement “I’m afraid of coronavirus more than anything else”, in the Polish version implies undefined fear without necessity compared with any point of reference. People with children feel more often uncomfortable when they think about the coronavirus (COVID-19). They are also significantly more worried about dying from COVID-19 infection, and they get upset when they hear about coronavirus in the media and on the Internet. The situation is similar when the respondents live with older people. The distribution of answers indicates that the respondents also experience physical symptoms that indicate anxiety, such as sweating palms.

Odds ratios were determined by regression analysis for the dependent variable emotional fear responses. The variables of gender, seniority, and relationship status are statistically insignificant.

For respondents who have children, we have determined a 2.4 odds ratio of an increase in the chance of a high level of anxiety (Table 5).

For the variable determining whether the tested person lives with the elderly, the odds ratios are statistically significant, and there is a 3-fold higher risk of emotional symptoms if one lives with an elderly person (Table 6).

The multiple model, which takes into account the simultaneous variable of having children and living with the elderly, is also statistically significant, but the odds ratios change very little. For all variants of the model, the absence of emotional symptoms is predicted, while the highest probability of occurrence of emotional symptoms has been ascertained for the situation when the examined person has children and lives with an elderly person.

The logistic regression model taking into account the physical variables and all seven anxiety symptoms did not show a statistically significant effect.

## 4. Discussion

In view of the strong influence of anxiety on individual and collective behavior, a scale for measuring fear was developed by a team of scientists to diagnose its level among people living in different parts of the globe. This study was conducted to assess the anxiety and fear of contracting the disease among dentists in Poland while working during the COVID-19 epidemic. It is very important because they are at a great risk of SARS-CoV-2 transmission via respiratory droplets [15,16]. Having defined a number of feelings which, according to the authors of the scale, make up the general characteristics of the level of anxiety, it can be concluded that the tested group was not subject to strong anxiety associated with COVID-19.

On average, the level of anxiety characterizing the studied population was 1.66 (ranging from 1—very low to 5—high). The obtained results are not consistent with the results of the study conducted in Poland on a group of 875 dentists, the aim of which was to assess the attitudes of dentists and their professional approach when confronted with the COVID-19 pandemic. In that study, more than half of the surveyed dentists expressed concerns about their own and their families’ health [17]. In this paper, over 70% of the respondents have suspended their practice, which was an effect resulting from insufficient availability of professional protective means, but it also resulted from the feelings of anxiety, uncertainty, and fear of the infection.

One of the main reasons for the discrepancy in anxiety levels among the population of Polish dentists between this study and the study conducted by Tysiąc-Miśta [17] could be the time when the survey was carried out. In this study, the survey-response period falls within the third month since the WHO announcement of the pandemic on 11 March 2020 (May–June 2020). In the case of the Tysiąc-Miśta study, it was the first month of the pandemic (April 2020). As indicated by Ferrara et al. in their study [18], the impact of the pandemic on anxiety, depression, and stress was highest in the first weeks of the pandemic and changed with time.

This is at variance with the limited professional activity that took place in the first months of the pandemic in Poland, when the vast majority of offices were closed. In April, a still rather high percentage of jobs did not resume work, and as late as in May, about 50% of private offices restarted their activity. It is also possible that the study was conducted at a time when the population managed to adapt better to the new situation, which to a lesser extent, reflects the primary emotions associated with the coronavirus. Undoubtedly, the dentists were dealing with adaptation to new working conditions that allow reducing the risk of transmission by using appropriate personal protective equipment (PPE) and complying with sanitary regulations. Moreover, a clear definition of the legal situation as to the potential risk of infection in the conditions of a dentist’s office appeared to have considerably calmed both the doctors and the patients themselves. As the study had been carried out among persons living in large cities, which are concurrently academic centers, we may assume that the relatively low anxiety level could arise among others from good access of the studied group of dentists to knowledge based on professional research. Good accessibility to library bases, direct information exchange between physicians, as well as contacts via professional online discussions make it possible for those persons to obtain reliable data quickly and easily, which as a consequence, helps minimize their anxiety. Furthermore, in large urban centers, dentists tend to work together in larger teams, which could also be considered to contribute to lower stress levels. It may also be assumed that mutual psychological support of fellow workers as well as the above-mentioned exchange of latest knowledge concerning the virus and ways of staying protected from the infection also minimized their anxiety. Additionally, dentists working in major clinics find it easier to organize work in a way that allows more convenient distribution of tasks (e.g., by adopting a shift system), which concurrently minimizes work overload and concerns. A further analysis of this issue appears to be quite an interesting venture.

Based on information obtained from the questionnaire, the concern about the health of the people for whom the respondents are responsible remains unchanged. Having children and caring for the elderly who are more susceptible to infections raises the level of anxiety in the respondents. It would seem that the presented attitude is irrelevant to the practiced profession, but it indicates the importance of basic family relations, especially in the period of danger. Results of studies published among physicians in Pakistan imply that there was a particularly high anxiety level found in women. According to the authors, this fact arises, apart from cultural reasons, from the responsibility for the family associated with their social functions of wives and mothers [19]. A similar dependence is observed in our own studies even though, without differentiation by gender, persons responsible for family members are exposed to anxiety and stress connected with the pandemic to a much greater extent. Perhaps medical education and knowledge in the field of epidemiology have a positive effect on the mental health of dentists. According to De Kock et al., systemic support, adequate knowledge, and resilience were identified as factors protecting against adverse mental health outcomes [20]. Additionally, results of answers given to the question concerning nervousness, namely “when I hear about the coronavirus in the media and the Internet”, seem to be quite interesting. In the situation of a virus that is invisible, we can deal with reactions intensified by the lack of proven knowledge and information chaos, as has been confirmed by our research. One in five respondents becomes nervous and anxious when they hear about the coronavirus (COVID-19) in the media and on the Internet. This can be further triggered by the frustration of being unable to divert attention away from the topic of the pandemic and the flood of information. Due to the scale of the problem, the World Health Organization (WHO) has added a “mythbusters” section to its website, where coronavirus-related advice is posted in order to debunk a large number of “fake news”. The study used a previously validated tool that has so far carried out thousands of assessments of anxiety levels around the world.

### The Limitations of the Study

The limitations of this study are that the data were collected within a short time period and, given the strong impact of the epidemic on the psychophysical sphere, it may be assumed that attitudes may evolve with the emerging new reports related to COVID-19. The examined sample corresponds to 0.9% of the total number of dentists, which is a relatively low percentage and may not be fully representative of the whole community. Moreover, the sampling method for the questionnaire was non-probabilistic. It was addressed to people from large academic centers, who have better access to both information and personal protective equipment. The use of social media could have also limited access to the study for elderly people who do not use a computer.

Additionally, the tool used does not take into account the current/active background for the study, which strongly affects the respondents’ responses. Depending on the emotional state in which they are examined and on objective circumstances, specific physiological reactions have been identified. Hence, statements concerning physiological responses, such as sweating of palms, sleeping problems, and an increased heart rate, are difficult to identify in a generalized perception of behavior-realistic information during catastrophic events [21]. In the event of a pandemic, the factors of anxiety are difficult to capture, which is another significant limitation for the obtained research results.

## 5. Conclusions

Studies concerning the anxiety level related to COVID-19 carried out among the Polish dentists ascertained that the tested level of anxiety among dentists was relatively low. However, the mindset, which is not easy to change, and the reaction to a continuous information flow concerning COVID-19 had a significant impact on the level of declared anxiety.

The COVID-19 Fear Scale (FCV- 19S), adjusted to the Polish language requirements, is a reliable tool that can be used effectively for analyzing the impact of any pandemic on the Polish-speaking population.

## Figures and Tables

**Table 1:** Demographic information.

Demographics	Group	n (%)
Gender	Female	245 (70.6%)
	Male	102 (29.4%)
Relationship status	Single	15 (4.3%)
	Divorced or widowed	52 (15.0%)
	In a relationship, living apart together	15 (4.3%)
	In a relationship, living together	38 (11.0%)
Having children	No	145 (41.8%)
	Yes	202 (58.2%)
Elderly care	No	297 (85.6%)
	Yes	50 (14.4%)

**Table 2:** Distribution of answers to individual questions of The COVID-19 Fear Scale.

Questionnaire Item	Group	Frequency	Percent	Mean	Std. Dev.
I am most afraid of COVID-19.	1	173	49.9	1.85	1.016
	2	85	24.5		
	3	63	18.2		
	4	21	6.1		
	5	5	1.4		
It makes me uncomfortable to think about COVID-19.	1	133	38.3	2.22	1.224
	2	85	24.5		
	3	68	19.6		
	4	43	12.4		
	5	18	5.2		
My hands become clammy when I think about COVID-19.	1	290	83.6	1.25	0.652
	2	36	10.4		
	3	14	4.0		
	4	5	1.4		
	5	2	0.6		
I am afraid of losing my life because of COVID-19.	1	250	72.0	1.47	0.897
	2	55	15.9		
	3	24	6.9		
	4	12	3.5		
	5	6	1.7		
When watching news and stories about COVID-19 on social media, I become nervous or anxious.	1	124	35.7	2.29	1.229
	2	87	25.1		
	3	64	18.4		
	4	57	16.4		
	5	15	4.3		
I cannot sleep because I’m worrying about getting COVID-19.	1	289	83.3	1.25	0.653
	2	37	10.7		
	3	14	4.0		
	4	5	1.4		
	5	2	0.6		
My heart races or palpitates when I think about getting COVID-19.	1	276	79.5	1.32	0.728
	2	44	12.7		
	3	18	5.2		
	4	6	1.7		
	5	3	0.9		

**Table 3:** Average percentage distribution of general anxiety defined by all marked feelings.

Level of Fear	Percent	Mean
1—Fear low	63.2	1.66
2	17.7	
3	10.9	
4	6.1	
5—Fear extremely high	2.1	

**Table 4:** Statistical significance of the chi-square test and values and statistical significance of Pearson’s correlation coefficient.

Questionnaire Item	Gender	Work Experience	Relationship Status	Having Children	Eldery Care
I am most afraid of COVID-19.	0.28	Pearson Correlation R = 0.12 Sig. = 0.03	0.42	0.00	0.01
It makes me uncomfortable to think about COVID-19.	0.08	Pearson Correlation R = 0.05 Sig. = 0.31	0.21	0.00	0.16
My hands become clammy when I think about COVID-19.	0.46	Pearson Correlation R = 0.01 Sig. = 0.83	0.58	0.41	0.00
I am afraid of losing my life because of COVID-19.	0.18	Pearson Correlation R = 0.12 Sig. = 0.03	0.38	0.02	0.26
When watching news and stories about COVID-19 on social media, I become nervous or anxious.	0.69	Pearson Correlation R = 0.10 Sig. = 0.08	0.36	0.00	0.00
I cannot sleep because I’m worrying about getting COVID-19.	0.28	Pearson Correlation R = 0.12 Sig. = 0.03	0.42	0.00	0.01
My heart races or palpitates when I think about getting COVID-19.	0.08	Pearson Correlation R = 0.05 Sig. = 0.31	0.21	0.00	0.16

**Table 5:** Model of logistic regression analysis of the variables emotional fear reactions and having children.

	b Coeff.	b Error	−95% CI	+95% CI	Wald Stat.	*p*-Value	Odds Ratio	−95% CI	+95% CI
Intercept	−2.36	0.45	−3.24	−1.47	27.32	<0.001	0.09	0.04	0.23
Having children	0.88	0.26	0.38	1.39	11.65	<0.001	2.42	1.46	4.02

**Table 6:** Model of logistic regression analysis of the variables emotional fear reactions and person lives with the elderly.

	b Coeff.	b Error	−95% CI	+95% CI	Wald Stat.	*p*-Value	Odds Ratio	−95% CI	+95% CI
Intercept	−2.20	0.39	−2.97	−1.44	32.02	<0.001	0.11	0.05	0.24
Person lives with the elderly	1.10	0.31	0.49	1.72	12.42	<0.001	3.01	1.63	5.57

## Data Availability

Data sharing not applicable No new data were created or analyzed in this study. Data sharing is not applicable to this article.
